# Genetic and cytological analyses reveal the recombination landscape of a partially differentiated plant sex chromosome in kiwifruit

**DOI:** 10.1186/s12870-019-1766-2

**Published:** 2019-04-30

**Authors:** S. M. Pilkington, J. Tahir, E. Hilario, S. E. Gardiner, D. Chagné, A. Catanach, J. McCallum, L. Jesson, L. G. Fraser, M. A. McNeilage, C. Deng, R. N. Crowhurst, P. M. Datson, Q. Zhang

**Affiliations:** 1 The New Zealand Institute for Plant and Food Research Limited (PFR), Private Bag 92169, Auckland, 1142 New Zealand; 2 PFR, Private Bag 11600, Palmerston North, 4442 New Zealand; 3 PFR, Private Bag 4704, Christchurch, 8140 New Zealand; 4 PFR, Private Bag 1401, Havelock North, 4157 New Zealand; 5 CAS Key Laboratory of Plant Germplasm Enhancement and Specialty Agriculture, Wuhan Botanical Garden, Chinese Academy of Sciences, Wuhan, 430074 China; 6 The Innovative Academy of Seed Design, Chinese Academy of Sciences, Wuhan, 430074 China

**Keywords:** Sex chromosome, Evolution, Sex determination, Kiwifruit, *Actinidia*, Recombination suppression

## Abstract

**Background:**

Angiosperm sex chromosomes, where present, are generally recently evolved. The key step in initiating the development of sex chromosomes from autosomes is the establishment of a sex-determining locus within a region of non-recombination. To better understand early sex chromosome evolution, it is important to determine the process by which recombination is suppressed around the sex determining genes. We have used the dioecious angiosperm kiwifruit *Actinidia chinensis* var*. chinensis*, which has an active-Y sex chromosome system, to study recombination rates around the sex locus, to better understand key events in the development of sex chromosomes.

**Results:**

We have confirmed the sex-determining region (SDR) in *A. chinensis* var. *chinensis*, using a combination of high density genetic mapping and fluorescent in situ hybridisation (FISH) of Bacterial Artificial Chromosomes (BACs) linked to the sex markers onto pachytene chromosomes. The SDR is a subtelomeric non-recombining region adjacent to the nucleolar organiser region (NOR). A region of restricted recombination of around 6 Mbp in size in both male and female maps spans the SDR and covers around a third of chromosome 25.

**Conclusions:**

As recombination is suppressed over a similar region between X chromosomes and between and X and Y chromosomes, we propose that recombination is suppressed in this region because of the proximity of the NOR and the centromere, with both the NOR and centromere suppressing recombination, and this predates suppressed recombination due to differences between X and Y chromosomes. Such regions of suppressed recombination in the genome provide an opportunity for the evolution of sex chromosomes, if a sex-determining locus develops there or translocates into this region.

**Electronic supplementary material:**

The online version of this article (10.1186/s12870-019-1766-2) contains supplementary material, which is available to authorized users.

## Background

Sex chromosomes have evolved independently in a number of plants [1, 2]. They have evolved from autosomes through the development of a sex-determining locus in a region of non-recombination or the development of non-recombination around the sex determining region [3], which blocks the exchange of genetic material between the sex chromosomes and enables them to begin to diverge from each other [4]. The combination of different selection pressures in each sex and the lack of recombination results in a range of evolutionary processes including Muller’s ratchet, background selection and genetic hitchhiking [5, 6].

Angiosperm sex chromosomes are much younger than mammalian sex chromosomes, which are estimated to have evolved approximately 167 million years ago (Mya) [7]. Mammalian Y chromosomes are generally severely degraded compared with their X chromosome counterparts, and contain few genes. This is thought to be a consequence of the lack of recombination between X and Y chromosomes in the male-specific region. The ancestor of the angiosperms was a hermaphrodite 140–200 Mya, and dioecy and sex chromosomes have evolved independently in different angiosperm families [3]. The sex chromosomes in *Silene latifolia* are estimated to have evolved 5–10 Mya [8] and those in papaya are also thought to have recently evolved through two chromosomal inversions occurring approximately 7.0 and 1.9 Mya [9]. The study of plant sex chromosomes can provide important insights into the evolution of sex chromosomes in general, especially the early stages [10, 11]. A key event in the formation of sex chromosomes in organisms that contain more than one sex determining gene is the suppression of recombination around these genes, which allows the multiple genes to be inherited as a single unit so that only male and female progeny are produced. Questions remain as to how this occurs [12, 13]. Studies of sex chromosomes in the early stages of evolution, such as those found in dioecious angiosperms, are needed to answer these questions.

The genus *Actinidia* (kiwifruit) contains more than 50 species of long-lived perennials [14]. All known *Actinidia* species are functionally dioecious, with male and female flowers on different plants [15]. *Actinidia* have a basic chromosome number of x = 29, many of the species are polyploid, ranging from diploid to octoploid including *A. chinensis* var. *chinensis,* which has diploid and tetraploid races [16]. Female plants bear flowers that appear complete; however, they produce only empty pollen grains, while flowers of male plants produce fertile pollen, but pistil growth is suppressed before style elongation or ovule initiation. Sex determination in *Actinidia* appears to be monofactorial at all ploidy levels, as male and female plants normally occur in a 1:1 ratio [17]. However, there are populations in which the inheritance of a Y chromosome is not 1:1 in the progeny, for example in populations where the male parent produces unreduced gametes [18]. Likewise, following the colchicine doubling of diploid males of *A. chinensis* var. *chinensis* to produce a tetraploid male (XXYY), male and female progeny are produced in a 5:1 ratio (Wu, unpublished). Rare ‘inconstant’ diploid male *A. chinensis* var. *deliciosa* plants that occasionally produce fruit have been identified; these fruiting males produce male and female progeny in a 3:1 ratio when selfed [19]. This indicates that in *Actinidia* there is a strict genetic control of sex expression by an active Y system, with diploids having homogametic females (XX) and heterogametic males (XY). Although there are no obvious dimorphic sex chromosomes in *Actinidia*, it has been suggested that in *A. chinensis* var. *chinensis,* the nucleolar organiser region (NOR)-containing chromosomes are the sex chromosomes [20]. This suggestion was based on observations of chromosome pairing during pachytene, where the satellite regions of the NOR-containing chromosomes paired in female plants, but did not pair in male plants. Genetic mapping of diploid *A. chinensis* var. *chinensis* also suggests that the sex-determining region (SDR) is located in the subtelomeric region of a single chromosome pair, linkage group 17 [21], which corresponds to the subtelomeric region of chromosome 25 [22]. It is not known how many genes are involved in sex determination in *Actinidia.*

As all extant *Actinidia* species are dioecious, regardless of the ploidy or species, it is very likely that their common ancestor was dioecious and contained sex chromosomes. The oldest fossil of the Actinidiaceae is suggested to be from 89 Mya [23] and the fossil record of *Actinidia* in China extends into the early Miocene or 20–26 Mya [24]; the dating of a backbone phylogeny of *Actinidia* suggested that the initial separation of the clades occurred *c.* 26.9 Mya [25]. The two other extant genera in the Actinidiaceae are *Saurauia* and *Clematoclethra*. *Saurauia* can be monoecious or functionally dioecious and *Clematoclethra* is monoecious [26], theoretically extending the upper limit of the age of the ancestral sex chromosomes in *Actinidia* into the Cretaceous, although it is likely that they have evolved much more recently than this. Compared with the sex chromosomes in mammals, sex chromosomes in *Actinidia* are in the early stages of their evolution.

To improve the identification and characterisation of the SDR in diploid *A. chinensis* var. *chinensis,* we have used high-density genetic mapping, microsatellite (SSR) mapping and genomic sequence data to investigate the recombination rates around the sex-determining locus. We have also identified hybridisation sites on pachytene chromosomes of Bacterial Artificial Chromosome (BAC) probes that we have found to be linked with the SDR.

## Results

### Recombination rates along pseudochromosome 25

**Fig. 1:**
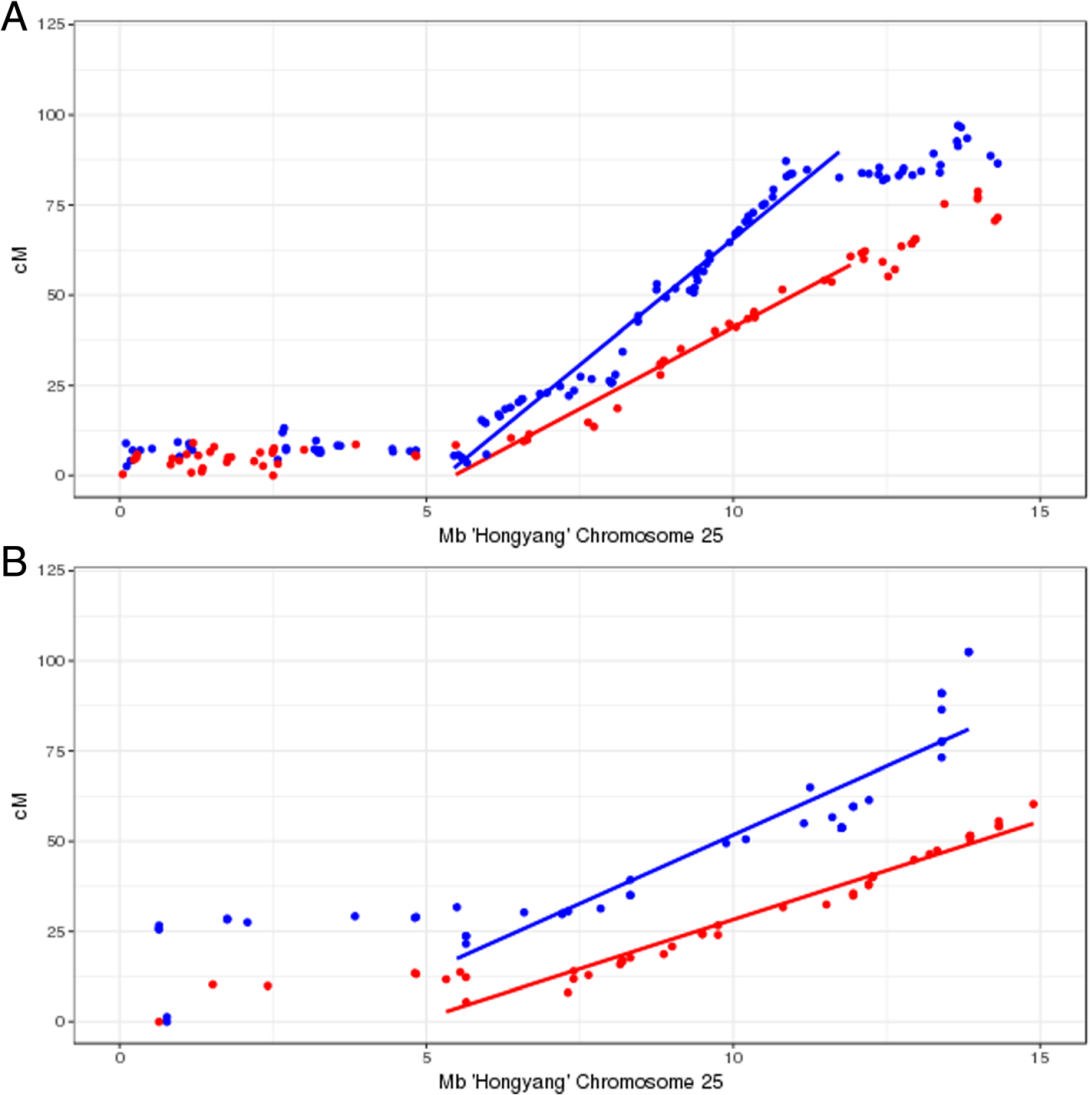
Recombination versus physical distance in **a** interspecific *Actinidia rufa* × *A. chinensis* var. *chinensis* (‘MT570001’ × ‘Guihai No4‘) mapping population I and **b** intraspecific *A. chinensis* var. *chinensis* (‘Hort16A’ × P1) mapping population II. Blue points and lines denote recombination rates in the male parent; red lines and points in the female parent

Recombination rates along *Actinidia* sex chromosomes (chromosome 25) as determined by recombinant mapping are shown in Fig. 1. The high density genetic map based on an interspecific F1 cross between *Actinidia rufa* ‘MT570001’ and *A. chinensis* var. *chinensis* ‘Guihai No4’ (Mapping population I) contains 210 markers along chromosome 25 [22], and exhibits suppressed recombination along the first 5–6 Mb of chromosome 25 in the *A. chinensis* var. *chinensis* (paternal) map, indicating minimal or no recombination between X and Y chromosomes in this region. The *A. rufa* (maternal) map also shows suppressed recombination in the terminal 5–6 Mb of chromosome 25, indicating minimal or no recombination between X chromosomes in this region. Likewise in the maternal and paternal *A. chinensis* var. *chinensis* genetic maps developed from ‘Hort16A’ × P1 (Mapping population II), suppressed recombination is observed over the terminal 5–6 Mb of chromosome 25. Overall recombination rates in the pseudoautosomal region were higher in the male parents of both mapping families (‘Guihai No 4’ 16.7 cM/Mb; P1 14.21 cM/Mb) than those in the females (‘MT57001’, 10.4 cM/Mb; ‘Hort16A’ 7.52 cM/Mb).

### Microsatellite mapping

**Fig. 2:**
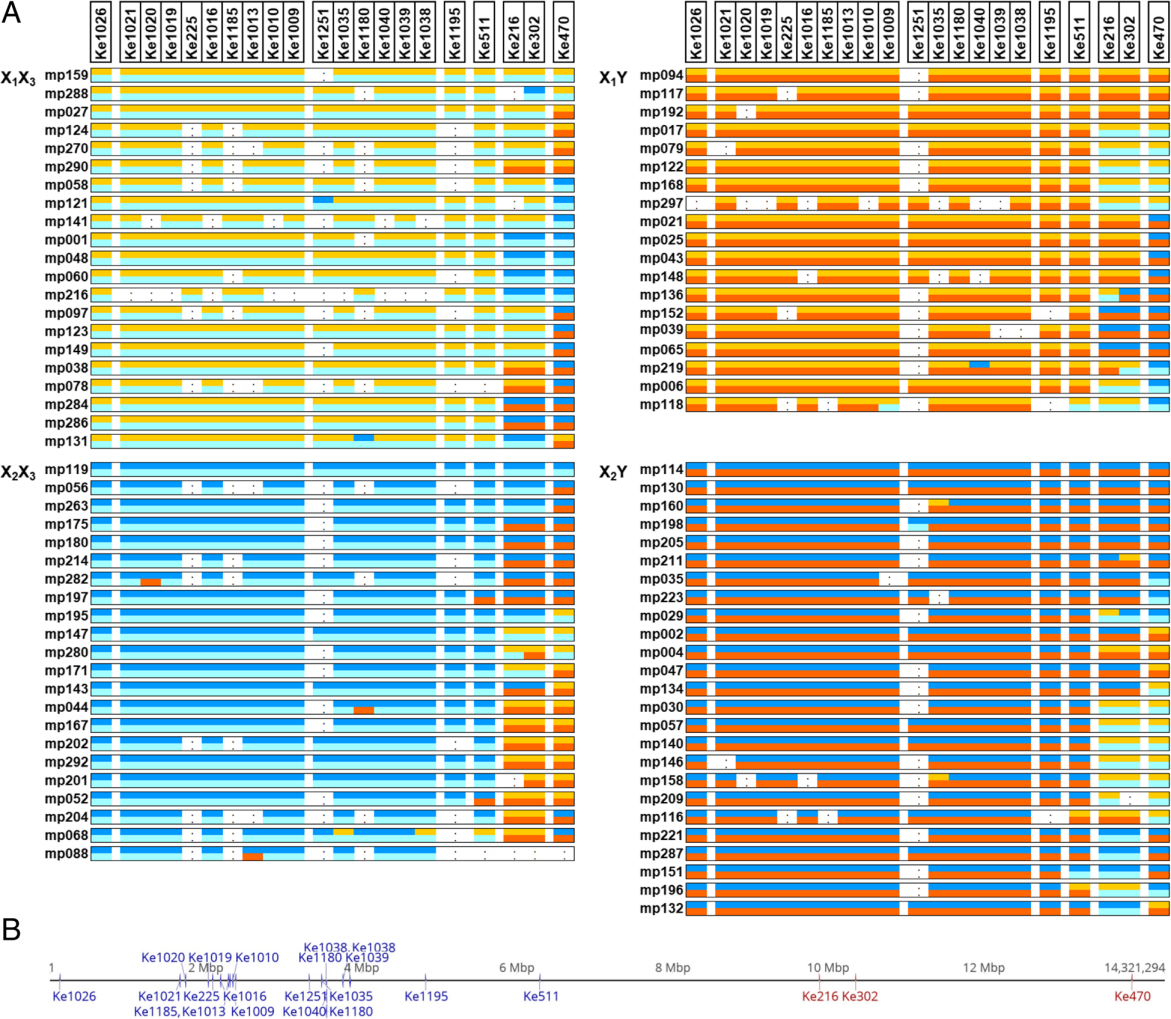
**a** Microsatellite markers were ordered by their position along *Actinidia chinensis* var. *chinensis* ‘Hongyang’ chromosome 25. For 87 members of the mapping population III (CK51_05 x CK15_02) [20], the chromosome of origin of each allele is shown (X_1_ (yellow), X_2_ (dark blue), X_3_ (light blue) or Y_1_ (red) or undetermined (.) and these are grouped by chromosome pairings. This was a consequence of using informative microsatellite markers, e.g. for fully informative marker Ke511 the parental alleles were a(217)b(205) and c(215)d(219) giving a(217)c(215) and b(205)c(215) female progenies and a(217)d(219) and b(205)d(215) male progenies, where the four alleles were detected as length differences in base pairs of amplified fragments during capillary electrophoresis **b** The marker positions on chromosome 25 of the ‘Hongyang’ genome are shown, with non-segregating markers in blue and segregating markers in red

Based on the inheritance of microsatellite marker alleles within the terminal 6 Mb of chromosome 25, the mapping population III progeny (an intraspecific diploid *A. chinensis* var. *chinensis* F1 mapping family from a cross between female parent X_1_X_2_ and male parent X_3_Y_1_) could be categorised into one of four groups: female progeny either X_1_X_3_ or X_2_X_3_ and male progeny either X_1_Y_1_ or X_2_Y_1_. The markers were ordered by their position in the ‘Hongyang’ genome, and the allelic content of microsatellites for these groups of progeny are shown in Fig. 2. For 18 of 21 markers, almost all the microsatellite alleles obtained were as expected for inheritance of this region from their parents with no recombination. However, as can be seen in Fig. 2, there were some exceptions, for example MP121 had a b allele for marker Ke1251 from chromosome X_2_ rather than an a allele from chromosome X_1_ as expected from the surrounding markers. Similarly in MP118 for marker Ke1009 there was a b allele from chromosome X_3_ rather than a c allele from the Y chromosome as expected from the surrounding markers.

### Distribution of annotations and genetic variation on Pseudochromosome 25

**Fig. 3:**
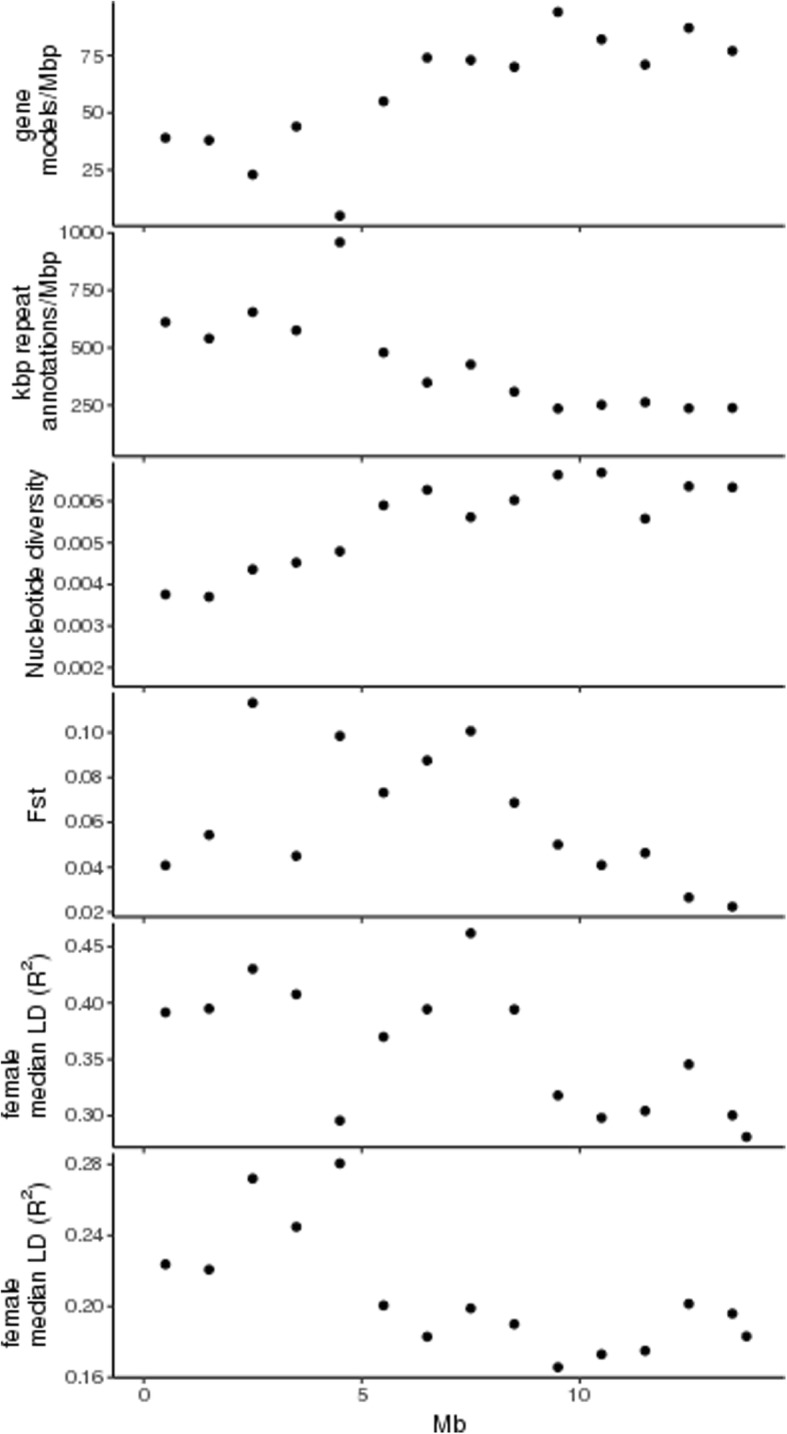
Distributions of annotations and genetic variation in 14 individuals of *Actinidia chinensis* var. *chinensis* (6 females and 8 males) determined in 1-Mb intervals of ‘Hongyang’ pseudochromosome 25. Median R^2^ denotes LD decay estimated as median R^2^ in the 1 kb–10 kb range for each 1 Mb bin

Inspection of the distribution of gene models and repeat annotations showed that the gene density is lower and repeat density higher in the terminal 0–6 Mb region than in the remainder of chromosome 25. A very gene-poor, repeat-rich region was identified at 4.5–5 Mb (Fig. 3). Genomic sequence data was available for 14 *A. chinensis* genotypes and these were used to calculate nucleotide diversity. Nucleotide diversity was found to be lower in the 0–5 Mb region than in the remainder of the chromosome. Windowed fixation index (Fst) measures of differentiation between males and females showed peaks in the 3–8 Mb region.

**Fig. 4:**
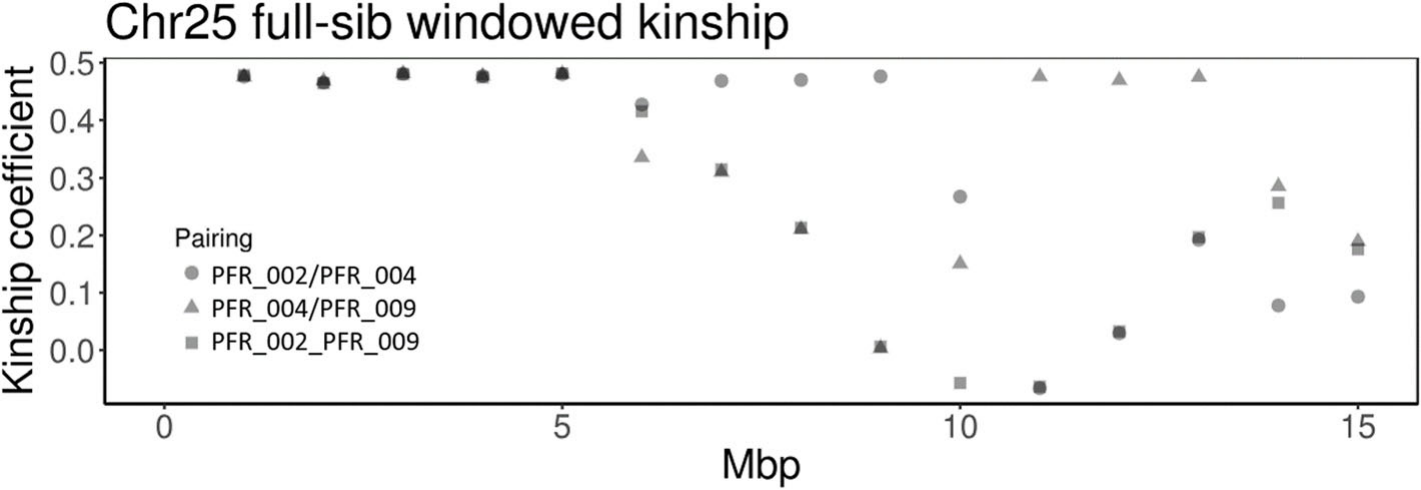
Windowed kinship of three full-sib male *Actinidia chinensis* var. *chinensis* within chromosome 25. Kinship coefficients were derived using the vcftools relatedness2 function in 1-Mb windows. Windows of pairings with 100% genetic identity are expected to have kinship coefficients of 0.5

To further assess meiotic recombination in *A. chinensis* var. *chinensis* sex chromosomes, we calculated kinship pairwise in 1-Mb windows between three full-sib males using whole genome sequence data. Each of the three full-sib males was expected to have received the same haplotype of the non-recombinant region of the Y chromosome from their paternal parent, and one of two possible X haplotypes from their maternal parent. Kinship between 0.46 and 0.48 was observed for the first 5 Mb of the pseudochromosome in all three full-sib-to-full-sib pairings, indicating highly similar to identical genotypes of this region in all three full-sib males (Fig. 4). Inheritance of the same X and Y haplotypes of this region reveals that they inherited the same X haplotype from their maternal parent (by chance, as each could theoretically have received either of the two haplotypes) and the same Y haplotype from their paternal parent through the entirety of the 5 Mb of the pseudochromosome. There was no recombination between the two X chromosomes or the X and Y chromosomes in this region during the meiosis that led to the production of these three full-sib males. For the rest of the chromosome, the windowed kinship coefficients ranged from zero or less (no haplotypes shared) to 0.47 (all haplotypes shared), consistent with recombination occurring throughout the rest of the chromosome.

### Fluorescent in situ hybridisation

**Fig. 5:**
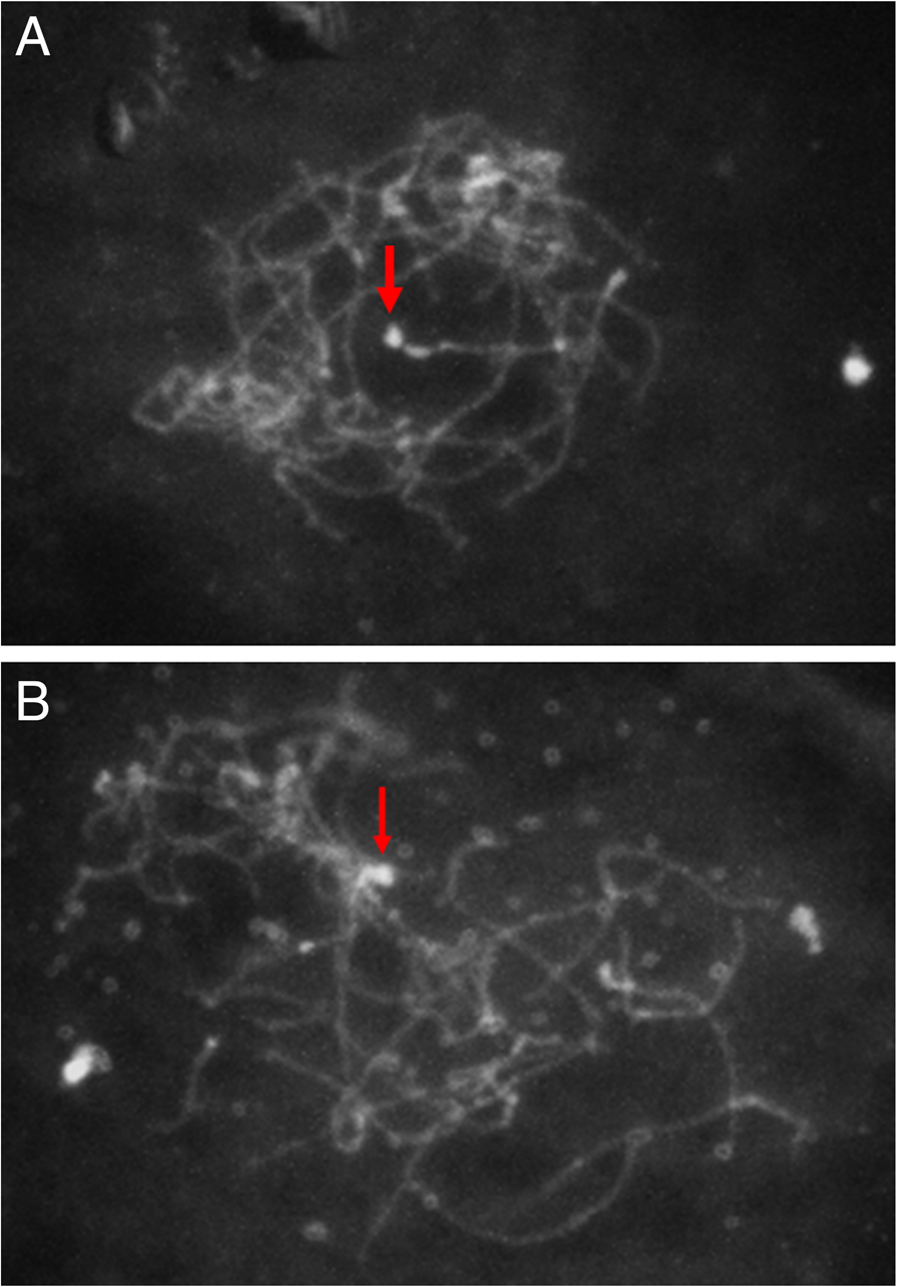
**a** Fluorescent in situ hybridisation on pachytene chromosomes of female *Actinidia chinensis* var. *chinensis* CK51_05 probed with BAC clone 47F17, which contains the sex-linked marker Ke225. The strong hybridisation signal present on the NOR-containing chromosome pair adjacent to the NOR is indicated by a red arrow. **b** Fluorescent in situ hybridisation on pachytene chromosomes of female *Actinidia chinensis* var. *chinensis* CK51_05 probed with BAC clone 180D13 which contains the sex-linked marker SmX, again strong hybridisation signal is seen at the terminal portion of the NOR-containing chromosome, indicated by a red arrow

The observed hybridisation patterns were similar for all three BAC probes derived from the SDR of the female parent. In the female *A. chinensis* var. *chinensis*, CK51_05, a strong hybridisation signal was observed at the terminal end of one chromosome pair, as well as some signals at centromeres (Fig. 5). The major hybridisation site was associated with a secondary constriction, identified as the NOR. The NOR accounts for approximately 5% of the total chromosome length in *A. chinensis* var. *chinensis* [20].

## Discussion

In our study we have employed several methods to characterise the recombination landscape of the SDR in *A. chinensis* var. *chinensis*, previously mapped genetically [21, 22]. In vitro fluorescent hybridisation using probes specific for the SDR indicated that a single chromosome pair was involved and furthermore, an analysis of recombination rates along chromosomes indicated the location and extent of suppressed recombination on X and Y variants of chromosome 25.

Recombination rates between the X and Y chromosomes were found to be very low in the terminal region of chromosome 25 where the SDR is located. Suppression of recombination was observed between X and Y chromosomes over the first 5–6 Mb in both male parents of the three bi-parental genetic mapping families studied. In addition, kinship analysis of three full-sib males showed that they all inherited the same haplotype from their paternal parent for the first 5 Mb of the Y chromosome. This is consistent with an active-Y type system, as sex determination is thought to be controlled by at least two tightly linked sex-determining genes on the Y chromosome, and any recombination between the sex-determining genes in male (XY) plants would produce recombinant asexual or hermaphroditic offspring, rather than the unisexual male and female offspring observed in *Actinidia*.

The suppressed recombination between X and Y chromosomes is regarded as the first step in the formation of sex chromosomes [27]. Recombination rates between the two X chromosomes in *A. chinensis* var. *chinensis* ‘Hort16A’ and those in *A. rufa* ‘MT570001’ were suppressed over the first 5–6 Mb. This is consistent with the hypothesis, as was the inheritance pattern for microsatellite markers, where the alleles originating from the same X haplotype were inherited together over the terminal 6 Mb. The kinship analysis of the three full-sib males showed that each inherited the same haplotype from their maternal parent for the first 5 Mb of the X chromosome, further evidence of suppression of recombination in this region. The similarities in the extent of the recombination suppression between the two X chromosomes, as well as between X and Y chromosomes, suggests that the recombination suppression in this region is not due to differences such as inversions between the X and Y chromosomes alone.

The similar pattern of recombination in both X and Y chromosomes of *A. chinensis* var. *chinensis*, of which 30–35% is a terminal non-recombining region where the sex-determining genes are located, while the remaining 65–70% undergoes normal recombination, suggests a mechanism for the evolution of sex chromosomes in *A. chinensis* var. *chinensis* from autosomes. Recombination rates are uneven across a genome, with both hotspots of recombination and regions of suppressed recombination [28]. This suppression of recombination around sex-determining genes is a key feature of sex chromosomes, allowing the two chromosomes of a pair to evolve separately. A key question in addressing how sex chromosomes are initially established is “how is recombination initially suppressed?” [29–31]. In *A. chinensis* var. *chinensis*, it appears that the SDR has evolved in a region of suppressed recombination because of the proximity of the NOR and centromere. The close proximity of the NOR and centromere to the sex-determining genes is likely to have created a region of suppressed recombination, allowing recombination to be restricted between the sex-determining genes, and has allowed sex chromosomes to evolve. In *A. chinensis* var. *chinensis* there are thought to be at least two sex-determining genes [32] and these would include the gene responsible for suppression of ovary development (*SuF*, according to the terminology of Westergaard M [33]), as well as a gene responsible for pollen maturation (*M*) [3]. The Y-encoded gene responsible for the suppression of ovary formation in *Actinidia* males has recently been identified as a male-specific type-C cytokinin response regulator [34]. The evolution of sex chromosomes requires not only evolution of alleles for these genes, but also for them to be located in a region of suppressed recombination, to enable the production of unisexual male and female offspring only [35].

Reduced rates of recombination around the centromere have been shown in several animals as well as in plants and fungi [36]. The inhibition of recombination around the centromere is important for the even segregation of chromosomes during meiosis. It is achieved through the *Ctf19*/*CCAN* kinetochore complex. It has been suggested that the complex suppresses recombination in two ways: inhibiting the formation of double strand breaks in the region close to the centromere, and recruiting cohesion to the pericentromeric region, which promotes inter-sister chromatid repair rather than inter-homologue repair [37]. There is also evidence for reduced rates of recombination around the ribosomal DNA (rDNA) in the NOR. Reduced rates of meiotic recombination along the NOR-containing chromosomes are observed in *Arabidopsis* [38], while in yeast, meiotic recombination between non-sister chromatids is strongly suppressed around the rDNA genes [39], although mitotic and meiotic recombination between sister chromatids occur frequently [39]. Low recombination rates in the SDR of *A. chinensis* var. *chinensis* are likely to be due to the proximity of both the NOR and centromere to the SDR. NORs are also present on the sex chromosomes in other organisms, for example *Drosophila* [40], plants such as liverwort [41] and spinach [42] and these NORs could have also played a role in suppressing recombination around sex-determining genes, allowing the establishment of sex chromosomes in these organisms.

The hybridisation patterns of sex-linked BAC probes indicated that the major hybridisation site was at the terminal region of the chromosome pair that contains a secondary constriction. The hybridisation signal spanned the terminal region, encompassing both the secondary constriction and the centromere (Fig. 5), suggesting that the SDR spans this region and that the sex-determining genes lie between these two features. As this was the only secondary constriction observed in *A. chinensis* var. *chinensis* chromosomes, it is likely to be the NOR. The presence of DNA repeats in the BACs which occur both on the sex chromosomes and some of the autosomes is the likely explanation for the hybridisation signal observed on other chromosomes. DNA repeats isolated from sex chromosomes of other plants have been demonstrated to be present on autosomes [43]. The FISH hybridisation results support He and colleagues’ [20] assertion that the NOR-containing chromosomes are the sex chromosomes (chromosome 25), and the telomeric position is consistent with genetic mapping results which show that the sex-determining locus is near the end of the linkage group corresponding to chromosome 25 [21, 22].

Although recombination was suppressed in the SDR, there was still a low level of genetic exchange between X chromosomes and between X and Y chromosomes. This was observed in the analysis of SSR inheritance in the SDR when some markers showed the presence of alleles that were not expected based on the surrounding markers. Two main possibilities would give rise to this pattern of inheritance: a double crossover event or gene conversion. Since a double crossover event would be extremely unlikely in this region because of suppressed recombination, this pattern is consistent with non-crossover gene conversion. A non-crossover gene conversion is the non-reciprocal transfer of short DNA segments between homologs. In the SDR region of *A. chinensis* var. *chinensis* there is evidence for X-X, X-Y and Y-X type gene conversions. For example, there is evidence of an X-X gene conversion at Ke1180 in MP131, evidence of an X-Y gene conversion was seen at Ke1009 in MP118, and evidence of a Y-X gene conversion was seen at Ke1013 in MP088. Overall, when considering the segregation of the markers within the terminal 5 Mb of chromosome 25, i.e. all markers apart from Ke511, Ke216, Ke302 and Ke470, there was evidence for four X1-X2 gene conversions (four unexpected alleles out of 559), three X2-X1 gene conversions (three unexpected alleles out of 495), two X3-Y gene conversions (two unexpected alleles out of 695) and three Y-X3 gene conversions (three unexpected alleles out of 638). Non-crossover gene conversions affect a short region of the DNA. The tracts have been estimated to span 50–1000 bp [44], and gene conversions are one of the more frequent genetic exchanges between homologs in plant genomes [45]. Inter-chromosomal gene conversions have been identified in the sex chromosomes of other organisms, including humans, primates, birds and mosses [46–49]. A combination of a lack of recombination and the occurrence of gene conversion would account for our results from recombination mapping, kinship analysis and SSR inheritance.

## Conclusions

The theory of sex chromosome evolution suggests that sex chromosomes were once an autosomal pair that evolved different morphology and gene content because they lost their ability to recombine [50]. The suppression of recombination around the SDR is a key step in this process. We have confirmed that in the sex chromosomes of *A. chinensis* var. *chinensis*, recombination is suppressed around the SDR between both X and Y and X and X chromosomes, suggesting that suppression of recombination in this region is not specific to differences between X and Y chromosomes. We propose that the proximity of the NOR and centromere to this region has created a region of suppressed recombination and that this, along with the evolution of male-sterile and female-sterile mutations, has enabled the sex chromosomes to evolve in *Actinidia*.

## Methods

### Plant material and DNA isolation

Three bi-parental mapping populations of *Actinidia* were used to study the recombination rates along chromosome 25. Mapping population I was an interspecific bi-parental mapping family *A. rufa* × *A. chinensis* var. *chinensis* (A, ‘MT570001’ × ‘Guihai No4’) [22]. Mapping population II was an intraspecific diploid *A. chinensis* var. *chinensis* family developed from a cross between ‘Hort16A’ and male parent P1. Mapping population III was also an intraspecific diploid *A. chinensis* var. *chinensis* family derived from a female from seed from Henan province, and the male parent from a seed accession from Guangxi province, China [21].

Seedlings from mapping population II were raised in tissue culture and 236 individuals selected for genotyping. Young expanded leaf tissue, weighing approximately 100 mg, was harvested and stored at − 80 °C by snap freezing in liquid nitrogen. Genomic DNA extraction was performed using the CTAB method [51]. DNA quantification was carried out using Qubit™ fluorometric analysis.

### Genotyping-by-sequencing (GBS), variant calling and selection of single nucleotide polymorphism (SNP) markers

The method for developing GBS libraries of population II followed [52], modified by the use of *Bam*HI for the restriction digestion step. The libraries were individually amplified and successful preparation verified by analysis of an aliquot by agarose gel electrophoresis, before pooling the amplicons prior to sequencing [53]. Libraries prepared from 236 genotypes were sequenced over 5 lanes using Illumina™ HiSeq2000 in single-end mode, with each lane generating more than 200 million single-end 100 bp reads. Two plates of libraries (192 genotypes) were sequenced over 2 lanes and half a plate (44 genotypes) was sequenced over 1 lane. SNP calling was performed using the reference-guided TASSEL pipeline on the Red5 (version PS1.1.68.5 [54], an earlier version of the published genome [55]) and the ‘Hongyang’ reference genomes [56]. SNPs were filtered at the filtering criteria of 0.7 for coverage across all genotypes, generating ~ 44–50 K SNP sites from each genome across 29 pseudochromosomes and unassigned scaffolds in linkage group Chr 30. SNP markers were de-convoluted for each parent using the following criteria. First, the SNP markers were selected that were heterozygous (ab) in one parent and homozygous (aa) in the other parent and vice versa. This generated a set of markers which would theoretically segregate as 1:1 < ab×aa> (pseudo-testcross) and are unique to each parent; these were used for construction of the male and female genetic linkage maps individually.

### Genetic map construction

Genetic maps using ‘Hort16A’ and P1 SNP markers were constructed using Joinmap3® (www.kyazma.nl). SNP marker data were processed in JoinMap using the ‘CP’ format for the population structure. Linkage groups were developed using default settings for grouping with modifications, including: a) the threshold range for Independence logarithm (base 10) of odds (LOD) started from a LOD score of 10 to 20 and, b) use of a regression mapping algorithm.

### Recombination rates along chromosomes

The physical positions of segregating SNP markers on the genetic map were plotted against physical SNP locations on pseudomolecules using R 3.3.0 (https://www.R-project.org) in two populations, an interspecific *Actinidia rufa* × *A. chinensis* var. *chinensis* (‘MT570001’ × ‘Guihai No4‘) mapping population I and an intraspecific *A. chinensis* var. *chinensis* (‘Hort16A’ × P1) mapping population II.

### Segregation of microsatellite alleles within the SDR

The inheritance pattern of SSR markers within the SDR was investigated in an intraspecific *A. chinensis* var. *chinensis* mapping population previously described [20]. Twenty-one microsatellite markers were selected for analysis; eighteen of these amplify from within the terminal 6 Mb of chromosome 25, the remaining three amplify from the distal portion. The parents and eighty-seven progeny of this mapping population were screened with these markers in the same manner as previously described [57]. The sequences of these primers and annealing temperatures are given in Additional file 1: Table S1. Based on the pattern of segregation of alleles from fully informative, female-informative and male-informative markers, alleles were grouped into one of four groups depending on the chromosome from which they had originated i.e. group 1 originated from chromosome X_1_, group 2 originated from chromosome X_2_ (inherited from the female parent), group 3 originated from X_3_, and group 4 from Y_1_ (inherited from the male parent).

### Whole-genome sequence alignment, variant calling and kinship analysis

Adaptors and low-quality or undetermined sequences of short-insert Illumina whole genome sequence were filtered and clipped using fastq-mcf (fastx toolkit, version 0.0.13) [58]. Paired reads at approximately 30x coverage were mapped to the ‘Hongyang’ genome [56] using bwa-mem 0.7.15 [59]. Variant calling was performed using Freebayes 1.1.0 [60], in 1-Mb windows. Variant Call Format (VCF) files were filtered using for biallelic variants with no missing data using the following vcflib (https://github.com/vcflib/vcflib) pipeline:

vcfbiallelic | vcffilter -f ‘NS = 14 & QUAL > 30 & SAR > 3 & PAIRED > 0.8 & SAF > 3.

VCF files were phased using Beagle 4.0 and default values [61]. Fst, Tajimas Pi and kinship coefficients were generated for each 1-Mb window of pseudochromosome 25 using vcftools 0.1.14 [62] and kinship determined using the vcftools relatedness2 option [63]. Rates of linkage disequilibrium (LD) decay was determined in 1-Mb windows using PopLDDecay (https://github.com/BGI-shenzhen/PopLDdecay). LD decay within each window was summarised as the median R^2^ in the 1 kb–10 kb sub-interval.

### Chromosome preparation

The female genotype of diploid *A. chinensis* var. *chinensis* used for this study, CK51_05, is the female parent of the mapping population III was used previously to construct a genetic map in *A. chinensis* var. *chinensis* [21]. Young flower buds at various stages were collected and placed immediately into 3:1 ethanol:acetic acid and stored at 4 °C for at least one day. If buds were to be stored for more than two weeks, they were transferred to 70% *v*/v ethanol and stored at − 20 °C until required.

Flower buds containing meiocytes at pachytene stage were identified by squashing an anther from each in FLP (formo:lacto:propiono) orcein and observing the meiotic stage. Once buds at pachytene had been identified, chromosome preparations were made following the method of Andras SC, Hartman TP, Marshall JA, Marchant R, Power JB, Cocking EC and Davey MR [64], using the remaining anthers in each bud and modified to use anthers rather than root tips. Anthers were hydrolysed in 1 M HCl at 37 °C for 15–20 min and then digested for 80 min in the following enzyme mixture: 4% (*w*/*v*) Onozuka R10 cellulase (Merck 102,321), 4% (w/v) cellulase (Sigma C-9442), 2% (w/v) pectoylase (Sigma P-3026) and 1% (w/v) cytohelicase (Sigma C-8274) dissolved in 0.01 M citrate buffer pH 4.5. The drop techniques of Felsenstein J [65] and Henegariu O, Heerema NA, Lowe Wright L, Bray-Ward P, Ward DC and Vance GH [66] were subsequently used to prepare chromosome spreads.

### Construction and screening of BAC library

The genomic DNA BAC library from *A. chinensis* var. *chinensis* CK51_05 was prepared by Bio S&T, Quebec, Canada and printed on 23 nylon filters in a 4 × 4 printing configuration. The genes *ndhA* (*NADH-dehydrogenase subunit A*) and *cox2* (*cytochrome c oxidase*) were employed to estimate contamination from chloroplasts and mitochondria, respectively. Only 0.6% of the BAC library contained organellar DNA. From a sample of 309 BAC clones, we determined the average insert size to be 71.32 ± 46.15 kb and that 30% of the sampled BAC clones contained large inserts of 80–260 kb.

Polymerase chain reaction (PCR) probes developed from a non-polymorphic marker derived from the sex linked SmX marker [67], and two genetic markers flanking the sex locus (Ke225 and udkac096) [21] were used to identify the SDR of the female parent. Purified PCR probes were labelled non-radioactively with digoxigenin-11-dUTP (Roche Diagnostics), hybridized at 65 °C overnight and detected as specified [68]. The corresponding BAC clones were isolated and the smallest clones for each of the three markers selected and labelled with biotin by nick-translation (Roche Diagnostics). These three clones were 47F17 - containing Ke225 (59.7 kb), 180D13 - containing SmX (49.3 kb), and 156B2 - containing udkac096 (85.2 kb).

### Fluorescent in situ hybridisation (FISH)

The FISH procedure followed a previously published method [64], with some modifications. Briefly, probes (50–100 ng) were dissolved in 2X SSCP (0.3 M NaCl, 0.03 M sodium citrate, 0.04 M sodium dihydrogen phosphate, pH 6.5), 50% formamide and 10% dextran sulphate and denatured at 85 °C for ten minutes. Thirty μL of this hybridisation mixture was added to each slide and the slide was covered with a plastic cover slip. Preparations were denatured for 5 min in 0.15 M NaOH in 70% ethanol and then dehydrated through an ice-cold ethanol series (70, 85, and 96% for 3 min each). Incubation was at 37 °C for 24 h prior to a wash in 2X SSC (0.3 M NaCl, 0.03 M sodium citrate) at 42 °C, followed by two stringent washes of 0.2X SSC for 15 min each at 42 °C (Stringency ~ 68%), and a final wash in detection buffer (0.1 M Tris, 0.15 M NaCl, pH 7.5) for 5 min at room temperature. The probe was detected using 50 ng/μL cy3-strepavidin conjugate (Sigma), 5% (*w*/*v*) bovine serum albumin (Sigma) in detection buffer for 60 min at 37 °C. Slides were then washed twice in detection buffer containing 0.05% (*v*/v) Tween® 20 for 15 min and the chromosome preparations were counter-stained for 5 min in 1 mM 4′,6-diamidino-2-phenylindole, dihydrochloride (DAPI) (Sigma) in 1X PBS pH 7.4 and mounted in 40 μL of mounting solution (0.2% (v/v) 1, 4-diazabicyclo-[2.2.2]octane (DABCO) (Sigma), 50% (v/v) glycerol in 1X PBS pH 7.4). Following storage at 4 °C for 2–3 days before observation, chromosome spreads were observed with an Olympus Vanox AHT3 light microscope using epi-fluorescence, and images were captured with an RS Photometrics CoolSNAP digital camera. Two images were captured per cell, at excitation wavelengths of 358 nm and 550 nm. Entire images were then manipulated using Adobe® Photoshop Version 6.0. Chromosome measurements were made using the computer application MicroMeasure version 3.3 [69].

## Additional file

Additional file 1: **Table S1**. Primer sequences for microsatellite analysis. (XLSX 16 kb)
